# Interleukin-35 Dampens CD8^+^ T Cells Activity in Patients With Non-viral Hepatitis-Related Hepatocellular Carcinoma

**DOI:** 10.3389/fimmu.2019.01032

**Published:** 2019-05-07

**Authors:** Lanlan Yang, Xue Shao, Shengnan Jia, Qian Zhang, Zhenjing Jin

**Affiliations:** Department of Hepatopancreatobiliary Medicine, The Second Hospital, Jilin University, Changchun, China

**Keywords:** interleukin-35, CD8^+^ T cells, non-viral hepatitis, hepatocellular carcinoma, immunotolerance

## Abstract

Interleukin (IL)-35 is a newly identified IL-12 cytokine family member, which has been demonstrated to induce immunotolerance by suppression of CD8^+^ T cells function in chronic viral hepatitis. However, the role of IL-35 in modulating CD8^+^ T cells activity in non-viral hepatitis-related hepatocellular carcinoma (HCC) was not fully elucidated. Forty-four patients with non-viral hepatitis-related HCC and 20 healthy individuals were enrolled. Serum IL-35 concentration was measured by ELISA. CD8^+^ T cells were purified from peripheral bloods and liver tissues. mRNA expression of cytotoxic/inhibitory molecules in CD8^+^ T cells with IL-35 stimulation was semi-quantified by real-time PCR. Direct and indirect contact co-culture systems of CD8^+^ T cells and HCC cell lines were set up. The modulatory function of IL-35 on peripheral and liver-resident CD8^+^ T cells was assessed by measurement of lactate dehydrogenase release and cytokine production in the co-culture supernatants. Serum IL-35 was notably elevated in HCC patients, while effective anti-tumor therapies down-regulated IL-35 concentration. Recombinant IL-35 stimulation suppressed cytotoxicity and proinflammatory cytokine secretion of peripheral and liver-resident CD8^+^ T cells in direct and indirect contact co-culture systems. This process was accompanied by reduction of perforin expression and interferon-γ production, as well as programmed death-1 and cytotoxic T-lymphocyte-associated protein 4 elevation in CD8^+^ T cells. The current data suggested that IL-35 inhibited both cytolytic and non-cytolytic function of CD8^+^ T cells to non-viral hepatitis-related HCC probably *via* repression of perforin expression. IL-35 might be considered to be one of the therapeutic targets for patients with HCC.

## Introduction

Hepatocellular carcinoma (HCC) is a major public health problem and one of the leading causes of cancer-related deaths all over the world (1). Although chronic hepatitis B virus (HBV) and hepatitis C virus (HCV) infection are the most pivotal risk factors for HCC in China, other non-viral liver disorder, including environmental borne carcinogens, alcoholism, and obesity, can also lead to HCC (2). A more recent study by Huang et al. indicated that metabolic risk factors (fatty liver, high triglyceride level, and diabetes mellitus history) are notably associated with non-viral HCC patients without alcoholism in endemic area of chronic hepatitis B (3). The liver plays an important immunoregulatory function through maintain immunotolerance, and non-parenchymal liver cells responsible for tolerogenic properties are the resident dendritic cells (DC) (4). Interaction between DC and other immune cells (CD4^+^ and CD8^+^ T cells) results in the immunologic homeostasis, and dysregulation of controlled immunologic network induces the hepatocarcinogenesis and development of HCC (5). However, the mechanism underlying this dysregulation, especially for non-viral hepatitis-related HCC, remains not completely understood.

Interleukin (IL)-35 comprises two heterodimeric subunits, IL-12 α chain p35 and IL-27 β chain Epstein-Barr virus-induce gene 3, and belongs to IL-12 cytokine family (6). IL-35 is the major effector cytokine secreted by multiple immunosuppressive subsets, including regulatory T cells (Tregs), regulatory B cells, and CD8^+^ Tregs (7), and exhibited suppressive activities in a range of infectious diseases, cancers, and autoimmune disorders (8, 9). IL-35 limited anti-tumor immunity via reduction of T cells proliferation and antigen-specific responses, decrease of long-term T cell memory and promotion of multiple inhibitory receptors (10). Fu et al. showed that higher expression of liver-resident IL-35 in HCC patients were more likely to suffer post-operative recurrence, and IL-35 was identified as an independent prognostic factor for recurrence free survival in HCC patients (11). In contrast, Long et al. showed that IL-35, which mainly localized in the cytoplasm of HCC cancer cells and peri-tumoral hepatocytes, revealed decreased expression in advanced stages (12). Our previous studies have indicated the immunosuppressive activity of IL-35 in chronic HBV and HCV infection by regulation of Tregs and CD8^+^ T cells (13, 14). Moreover, IL-35 did not affect the bioactivity of HBV antigen-expressing HepG2.2.15 cells and non-HCV infected Huh7.5 cells (13, 14). However, the role of IL-35 in regulation of CD8^+^ T cells function in HCC was still not fully elucidated. Thus, we functionally analyzed the purified CD8^+^ T cells activity from non-viral hepatitis-related HCC in response to recombinant IL-35 stimulation *in vitro*.

## Materials and Methods

### Enrolled Subjects

A total of 44 patients with pathologically diagnosed, non-viral hepatitis-related HCC were enrolled in the current study, and all patients were hospitalized in The Second Hospital of Jilin University from March 2014 to July 2017. Blood samples were collected from all enrolled patients, while fresh HCC specimens and non-tumor site liver specimens were obtained from HCC patients who underwent surgery in The Second Hospital of Jilin University. No patients received chemotherapy, radiotherapy, or immunomodulatory therapy before baseline sampling and prior to surgery. No patients were chronically infected with viral hepatitis or afflicted with immune disorders. The stage of tumor was determined according to Barcelona Clinic Liver Cancer (BCLC) staging system. For normal controls (NC), 26 sex- and age-matched healthy individuals were also enrolled. The baseline characteristics of enrolled subjects were shown in Table 1. This study was carried out in accordance with the recommendations of Ethics Committee of The Second Hospital of Jilin University with written informed consent from all subjects. All subjects gave written informed consent in accordance with the Declaration of Helsinki. The protocol was approved by the Ethics Committee of The Second Hospital of Jilin University.

**Table 1:** Clinical characteristics of enrolled subjects.

	**NC**	**HCC**
Cases (*n*)	20	44
Gender (male/female)	13/7	31/13
Age (years)	39.2 ± 9.0	45.8 ± 13.1
AFP (ng/ml)	< 20 (3.18~13.41)	>400 (401.7~120,500)
BCLC stage (A/B/C/D)	Not available	17/11/9/7
Underlying liver disease		
Non-viral hepatitis-related cirrhosis	Not available	16
Unknown	Not available	28

### Peripheral Blood Mononuclear Cells (PBMCs) and Intrahepatic Lymphocytes (IHLs) Isolation

PBMCs were isolated by density gradient centrifugation using Ficoll-Hypaque (Sigma-Aldrich, St Louis, MO, USA). For IHLs isolation, fresh liver specimen was diced into small pieces in 5 mL RPMI1640, and were passed through a fine steel sieve. The resulting suspension were incubated at 37°C for 30 min in RPMI1640 supplemented with 10% of heat-inactivated fetal bovine serum (FBS), 0.5 mg/mL of collagenase V, and 0.001% of DNase I. Cultured suspension were added into 40 mL of cold, serum free RPMI1640, and were centrifuged at 16 × g for 2 min at 16°C. The supernatants were harvested and were centrifuged at 300 × g for 10 min at 4°C. The pellets were resuspended in 3 mL sterile 44% (*v/v*) Percoll (Sigma-Aldrich) in RPMI1640. The solution were layed over 5 mL 56% (*v/v*) Percoll in PBS, and then were gradiently centrifuged at 850 × g for 30 min at 20°C. IHLs were harvested and washed with PBS twice by centrifugation at 400 × g for 10 min at 4°C, and were cultured in RPMI1640 supplemented with 10% of FBS at 37°C under 5% CO_2_ condition.

### CD8^+^ T Cells Purification

CD8^+^ T cells were purified using human CD8^+^ T Cell Isolation Kit (Miltenyi, Bergisch Gladbach, Germany) according to the instructions from the manufacturer. The purity of enrich cells was more than 95% by flow cytometry determination.

### Cell Culture

Two different HCC cell lines, HepG2 cells and HLA-A2 expressing plasmid (15) stably transfected Huh7 cells, were cultured in DMEM supplemented with 10% of FBS, 100 U/L of penicillin, and 0.1 mg/mL of streptomycin at 37°C under 5% CO_2_ condition. CD8^+^ T cells purified from HLA-A2 restricted HCC patients were stimulated with recombinant IL-35 (Peprotech, Rocky Hill, NJ, USA; final concentration 1 ng/mL) for 6 h, as previously described (13, 14). Cells were washed twice, and were co-cultured in direct contact and in indirect contact with target HepG2 or HLA-A2-expressing Huh7 cells (ratio of effector cells to target cells = 1: 5) in the presence of anti-CD3/CD28 (eBioscience, San Diego, CA, USA) or HLA-A2 restricted peptide derived from human alpha fetoprotein (AFP) (sequence FMNKFIYEI; final concentration 10 μg/mL) (16, 17) for 48 h as previously described (14, 18). In direct contact co-culture system, CD8^+^ T cells and target cells were directly mixed together in 24-well plates. In indirect contact co-culture system, CD8^+^ T cells were added into upper chamber, while target cells were seeded into lower chamber of Transwell plate (Corning, Corning, NY, USA). Thus, CD8^+^ T cells and target cells were separated by a 0.4 μm membrane, which allowed passage of soluble factors only. Supernatants and cultured cells were harvested for further experiments.

### Enzyme Linked Immunosorbent Assay (ELISA)

IL-35 and interferon (IFN)-γ expression was measured using commercial ELISA kits (CUSABIO, Wuhan, Hubei Province, China) according to the instructions from the manufacturer.

### Cytokine Assay

The following cytokines levels in the cultured supernatants, including IFN-γ, IL-1β, IL-10, IL-12p70, IL-6, IL-8, and tumor necrosis factor (TNF)-α, were measured using Human Proinflammation 7-Plex Base Kit (Meso Scale Discovery, Rockvillie, MD, USA) by SECTOR Imager (Meso Scale Discovery) according to the instructions from the manufacturer.

### Proliferation Assay

Cellular proliferation was measured using Cell Counting Kit-8 (CCK-8, Beyotime, Wuhan, Hubei Province, China) according to the instructions from the manufacturer.

### Real-Time PCR

Total RNA was isolated from CD8^+^ T cells using Trizol reagent (Invitrogen, Carlsbad, CA, USA) according to the instructions from the manufacturer. cDNA was synthesized with random hexamers using PrimeScript RT Master Mix (TaKaRa, Dalian, Liaoning Province, China). Real-time PCR was performed using SYBR Premix Ex *Taq* (TaKaRa). The relative gene expression was quantified using 2^−Δ*ΔCT*^ method with ABI7500 System Sequence Detection software (Applied Biosystems, Foster, CA, USA). The primers sequences were used as previously described (19).

### Flow Cytometry

Purified CD8^+^ T cells with or without IL-35 stimulation were incubated in the presence of anti-CD8 APC Cy7 (eBioscience) and anti- programmed death-1 (PD-1) FTIC (eBioscience) for surface staining, and anti- cytotoxic T-lymphocyte-associated protein 4 (CTLA-4) PE (eBioscience) for intracellular staining. In certain experiments, purified CD8^+^ T cells were stimulated with either PMA (50 ng/mL)+ionomycin (1 μg/mL) or AFP peptide in the presence of monensin (10 μg/mL) for 6 h. Cells were transferred to FACS tubes, and anti-CD8 APC Cy7 (eBioscience) was added for a 20 min incubation at 4°C in the dark. Cells were then stained with anti-IFN-γ APC (eBioscience) for 20 min at room temperature after fixation and permeabilization. Isotype controls were used to enable correct compensation and confirm antibody specificity. Acquisitions were performed using Cell Quest Pro Software (BD Biosciences Immunocytometry Systems, San Jose, CA, USA) in a FACS Calibur analyser (BD Biosciences Immunocytometry Systems). Data were analyzed using FlowJo Software Version 8.4.2 for Windows (Tree Star, Ashland, OR, USA).

### Cytotoxicity of Target Cells

The cytotoxicity of target HepG2 or Huh7 cells was assessed by measuring lactate dehydrogenase (LDH) expression in the cultured supernatants at the end of incubation period using LDH Cytotoxicity Assay Kit (Beyotime) according to the instructions from the manufacturer. LDH expression in HepG2 cells or HLA-A2-expressing Huh7 cells was determined as low-level control, while LDH expression in Triton X-100-treated, HepG2 cells or HLA-A2-expressing Huh7 cells was determined as high-level control. The percentage of cell death was calculated by the following equation: (experimental value - low-level control)/(high-level control - low-level control) × 100%.

### Statistical Analyses

All data were analyzed using SPSS19.0 for Windows (SPSS, Chicago, IL, USA). Shapiro-Wilk test was used for normal distribution assay, and all variables were following normal distribution. Data were presented as mean±standard deviation, and statistical significance was determined by Student *t*-test, SNK-*q* test, or paired *t*-test. Values of *P* < 0.05 were considered as significant differences.

## Results

### Serum IL-35 Level Was Elevated in Patients With Non-viral Hepatitis-Related HCC

**Figure 1:**
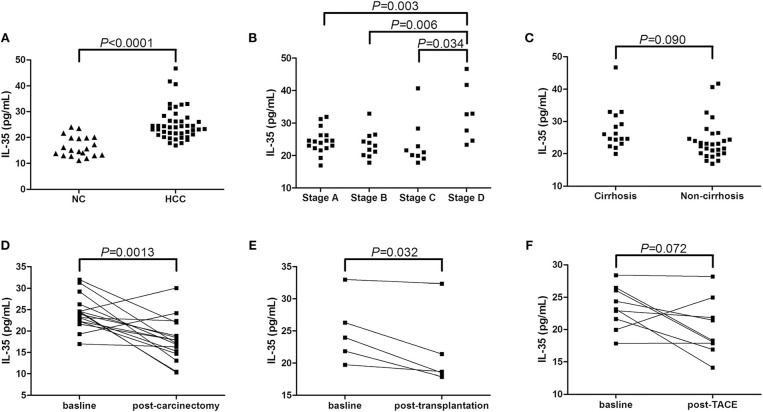
IL-35 expression in non-viral hepatitis-related hepatocellular carcinoma. **(A)** The concentration of IL-35 in the serum was measured by ELISA in healthy individuals (*n* = 20) and non-viral hepatitis-related HCC (*n* = 44). The individual level for each subject was shown. Significance was assessed using Student *t*-test. **(B)** Serum IL-35 level was compared among HCC patients in BCLC stage A (*n* = 17), stage B (*n* = 11), stage C (*n* = 9), and stage D (*n* = 7). Significances were assessed using SNK-*q* test. **(C)** Serum IL-35 was also compared between patients with cirrhosis (*n* = 16) and without cirrhosis (*n* = 28). Significance was assessed using Student *t*-test. **(D)** IL-35 expression in the serum was measured in patients underwent hepatic carcinectomy (*n* = 16). Significance between baseline and post-carcinectomy was assessed using paired *t*-test. **(E)** Serum IL-35 expression was measured in patients underwent liver transplantation (*n* = 5). Significance between baseline and post-transplantation was assessed using paired *t*-test. **(F)** Serum IL-35 level was also measured in patients underwent TACE (*n* = 9). Significance between baseline and post-operation was assessed using paired *t*-test.

We firstly screened IL-35 expression in the serum in non-viral hepatitis-related HCC patients. Serum IL-35 was increasingly expressed in HCC patients compared with in healthy individuals (25.36 ± 6.37 pg/mL vs. 16.52 ± 3.95 pg/mL, Student *t-*test, *P* < 0.0001, Figure 1A). IL-35 expression in the serum was also elevated in BCLC stage D patients (32.85 ± 8.72 pg/mL) in comparison with stage A (24.47 ± 3.84 pg/mL, SNK-*q* test, *P* = 0.003, Figure 1B), stage B (23.45 ± 4.15 pg/mL, SNK-*q* test, *P* = 0.006, Figure 1B), and stage C patients (23.53 ± 7.12 pg/mL, SNK-*q* test, *P* = 0.034, Figure 1B). However, there was no statistical difference of serum IL-35 expression between patients with cirrhosis and without cirrhosis (27.50 ± 6.47 pg/mL vs. 24.13 ± 6.09 pg/mL, Student *t-*test, *P* = 0.090, Figure 1C). Furthermore, Twenty-one HCC patients (all 17 patients in stage A and 4 patients in stage B) underwent hepatic carcinectomy (*n* = 16) or liver transplantation (*n* = 5). Serum samples were collected 1 or 2 months after surgery. Patients who underwent hepatic carcinectomy received routine therapy post-operation, and serum IL-35 was down-regulated post-carcinectomy (24.36 ± 3.94 pg/mL vs. 17.88 ± 5.04 pg/mL, paired *t-*test, *P* = 0.0013, Figure 1D). Patients who underwent liver transplantation received methylprednisolone therapy immediate post-operation, followed by immuno-suppressants (tacrolimus plus mycophenolate mofetil) therapy 3 days post-transplantation, and IL-35 expression in the serum was also reduced 1 month post-transplantation (24.97 ± 5.10 pg/mL vs. 21.76 ± 6.08 pg/mL, paired *t-*test, *P* = 0.032, Figure 1E). Other nine patients (5 in stage B and 4 in stage C) underwent transcatheter arterial chemoembolization (TACE), and serum were also collect 1 or 2 months post-TACE. There was a decreasing trend of IL-35 level in the serum post-TACE, however, this difference failed to achieve statistical significance (23.43 ± 3.33pg/mL vs. 20.19 ± 4.36 pg/mL, paired *t-*test, *P* = 0.072, Figure 1F).

### IL-35 Stimulation Inhibited Perforin mRNA Expression and IFN-γ Production in CD8^+^ T Cells From Non-viral Hepatitis-Related HCC

**Figure 2:**
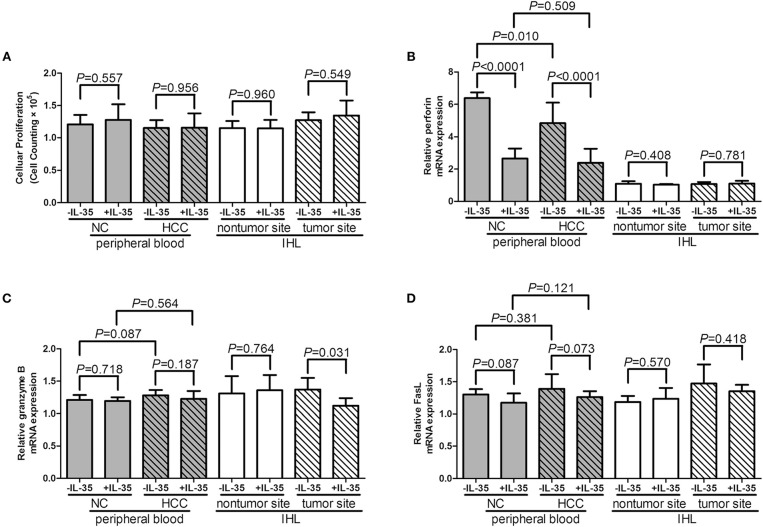
The influence of IL-35 on proliferation and cytotoxic pathways of peripheral and liver-resident CD8^+^ T cells from healthy individuals and non-viral hepatitis-related hepatocellular carcinoma. CD8^+^ T cells were purified from peripheral bloods of NC (*n* = 6) and HCC patients (*n* = 13) as well as liver specimens from non-tumor site and tumor site (HLA-A2 restricted, *n* = 5). 10^5^ of purified CD8^+^ T cells were stimulated with recombinant IL-35 for 6 h. **(A)** CD8^+^ T cells proliferation in response to IL-35 stimulation was assessed by CCK-8 methods. mRNA expression corresponding to **(B)** perforin, **(C)** granzyme B, and **(D)** FasL in CD8^+^ T cells with or without IL-35 stimulation was measured by real-time PCR. The columns indicated means, and the bars indicated standard deviation. Significances were determined using Student *t*-test or paired *t*-test.

Our previous study has revealed that IL-35 did not affect bioactivity of HBV antigen expressing HCC cell line HepG2.2.15 cells, which indicated that IL-35 might mainly contribute to immune cells function (14). Thus, we investigated the influence of IL-35 on bioactivity of CD8^+^ T cells in patients with non-viral hepatitis-related HCC. CD8^+^ T cells were purified from peripheral bloods of healthy individuals (*n* = 6) and HCC patients (*n* = 13) as well as liver specimens from non-tumor site and tumor site (HLA-A2 restricted, *n* = 5). 10^5^ of purified CD8^+^ T cells were stimulated with recombinant IL-35 for 6 h. CCK-8 results revealed that IL-35 stimulation did not affect CD8^+^ T cells proliferation either from peripheral bloods or liver specimens (paired *t*-tests, all *P* > 0.05, Figure 2A). Cytokine production in the cultured supernatants was also measured, however, there were no remarkable differences of cytokines production by CD8^+^ T cells in the presence or absence of IL-35 stimulation (Tables 2, 3). Furthermore, mRNA expression corresponding to perforin, granzyme B, and FasL was measured within CD8^+^ T cells in response to IL-35 stimulation. Perforin mRNA expression was elevated in peripheral CD8^+^ T cells from healthy individuals than those from HCC patients (Student *t-*test, *P* = 0.010, Figure 2B). IL-35 stimulation notably down-regulated perforin mRNA expression in peripheral CD8^+^ T cells from both NC and HCC patients (paired *t*-tests, all *P* < 0.0001, Figure 2B). However, there were no significant differences of perforin mRNA expression between liver-resident CD8^+^ T cells from non-tumor site and those from tumor site (Student *t-*test, *P* > 0.05, Figure 2B) or between prior to and post IL-35 stimulation (paired *t*-tests, *P* = 0.408 and *P* = 0.781, Figure 2B). There were also no remarkable differences of either granzyme B (Figure 2C) or FasL (Figure 2D) mRNA expression in peripheral and liver-resident CD8^+^ T cells (Student *t-*tests, all *P* > 0.05). IL-35 stimulation only reduced granzyme B mRNA expression in liver-resident CD8^+^ T cells from tumor site (paired *t*-test, *P* = 0.031, Figure 2C), however, did not affect FasL mRNA expression (paired *t*-tests, all *P* > 0.05, Figure 2D).

**Table 2:** Cytokine production by peripheral CD8^+^ T in response to IL-35 stimulation (pg/mL).

	**NC (*****n*** **=** **6)**			**HCC (*****n*** **=** **13)**		
	**–IL-35**	**+IL-35**	***P*-value**	**–IL-35**	**+IL-35**	***P*-value**
IFN-γ	13.57 ± 4.82	14.77 ± 5.18	0.419	11.75 ± 3.46	12.72 ± 2.43	0.564
IL-1β	37.79 ± 3.54	34.43 ± 8.90	0.229	41.35 ± 9.69	39.11 ± 4.98	0.642
IL-10	N.D.	N.D.	–	N.D.	N.D.	–
IL-12p70	61.97 ± 18.98	53.88 ± 21.21	0.091	39.86 ± 9.73	32.67 ± 7.61	0.239
IL-6	71.45 ± 7.00	70.64 ± 11.13	0.332	58.48 ± 8.39	48.87 ± 14.19	0.157
IL-8	57.64 ± 16.07	51.58 ± 21.61	0.344	47.75 ± 8.56	46.64 ± 11.42	0.299
TNF-α	1527 ± 288.8	1346 ± 224.4	0.163	1066 ± 349.8	1059 ± 163.2	0.106

*N.D., not detected. P-values were determined using paired t-tests*.

**Table 3:** Cytokine production by liver-resident CD8^+^ T in response to IL-35 stimulation (pg/mL).

	**Non-tumor site (*****n*** **=** **5)**			**Tumor site (*****n*** **=** **5)**		
	**–IL-35**	**+IL-35**	***P*-value**	**–IL-35**	**+IL-35**	***P*-value**
IFN-γ	13.36 ± 2.68	14.78 ± 4.87	0.136	16.32 ± 4.69	12.35 ± 1.78	0.634
IL-1β	43.80 ± 9.79	37.35 ± 18.72	0.472	49.55 ± 7.84	47.53 ± 8.66	0.751
IL-10	N.D.	N.D.	–	N.D.	N.D.	–
IL-12p70	67.61 ± 15.33	64.37 ± 24.79	0.301	44.18 ± 15.93	47.82 ± 9.34	0.882
IL-6	77.87 ± 27.50	74.84 ± 26.16	0.879	54.68 ± 8.96	57.48 ± 10.63	0.837
IL-8	66.74 ± 18.37	59.42 ± 11.96	0.119	56.36 ± 6.86	57.61 ± 10.89	0.812
TNF-α	1561 ± 267.2	1337 ± 342.6	0.525	1390 ± 216.6	1356 ± 351.8	0.267

*N.D., not detected. P-values were determined using paired t-tests*.

**Figure 3:**
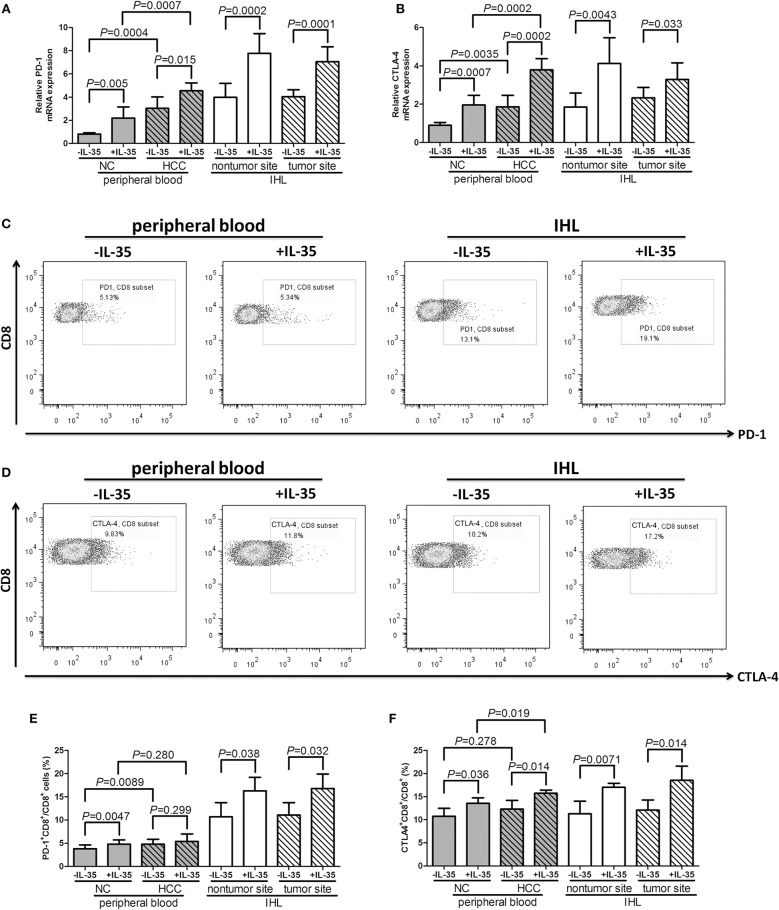
The influence of IL-35 on PD-1 and CTLA-4 expression of peripheral and liver-resident CD8^+^ T cells from healthy individuals and non-viral hepatitis-related hepatocellular carcinoma. CD8^+^ T cells were purified from peripheral bloods of NC (*n* = 6) and HCC patients (*n* = 13) as well as liver specimens from non-tumor site and tumor site (HLA-A2 restricted, *n* = 4). 10^5^ of purified CD8^+^ T cells were stimulated with recombinant IL-35 for 6 h. mRNA expression corresponding to **(A)** PD-1 and **(B)** CTLA-4 in CD8^+^ T cells with or without IL-35 stimulation was measured by real-time PCR. Representative flow dots of **(C)** PD-1 and **(D)** CTLA-4 expression in CD8^+^ T cells prior to and post IL-35 stimulation were shown. **(D)** Representative flow dots of CTLA-4 expression in CD8^+^ T cells prior to and post IL-35 stimulation were shown. Percentage of **(E)** PD-1^+^CD8^+^ cells and **(F)** CTLA-4^+^CD8^+^ cells was compared prior to and post IL-35 stimulation. The columns indicated means, and the bars indicated standard deviation. Significances were determined using Student *t*-test or paired *t*-test.

Furthermore, mRNA expressions and protein levels for exhausted markers, programmed death-1 (PD-1) and cytotoxic T-lymphocyte-associated protein 4 (CTLA-4), were also assessed in CD8^+^ T cells. IL-35 stimulation notably elevated PD-1 mRNA (paired *t*-tests, all *P* < 0.05, Figure 3A) and CTLA-4 mRNA (paired *t*-tests, all *P* < 0.05, Figure 3B) expression in both peripheral and liver-resident CD8^+^ T cells. PD-1 and CTLA-4 protein expression in CD8^+^ T cells in response to IL-35 stimulation was also measured by flow cytometry. The representative flow dots of PD-1 and CTLA-4 expression in CD8^+^ T cells prior to and post IL-35 stimulation were shown in Figures 3C,D, respectively. PD-1 expression in liver-resident CD8^+^ T cells was increased than in peripheral bloods (Figure 3E). The protein expression pattern was similar to mRNA levels following IL-35 stimulation. PD-1^+^CD8^+^ T cell percentage was elevated in HCC patients when compared with healthy individuals (Student *t-*tests, *P* = 0.0089, Figure 3E), however, CTLA-4^+^CD8^+^ T cells percentage was comparable between patients and controls (Student *t-*tests, *P* = 0.278, Figure 3F). IL-35 stimulation promoted PD-1 and CTLA-4 expression in CD8^+^ T cells in both peripheral and liver-resident CD8^+^ T cells (paired *t*-tests, *P* < 0.05, Figures 3E,F) expect in peripheral bloods from HCC patients (paired *t*-tests, *P* = 0.299, Figure 3E).

**Figure 4:**
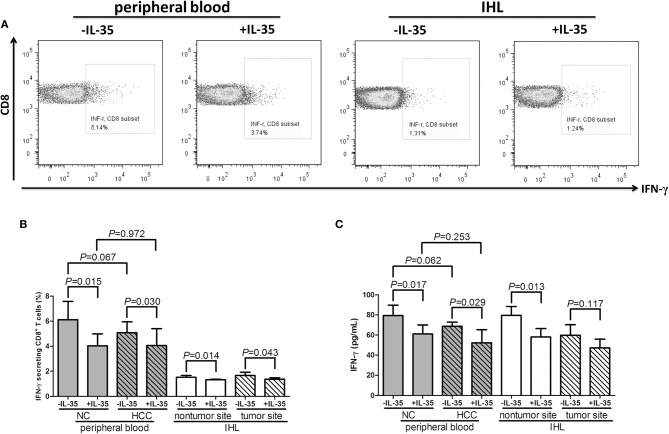
The influence of IL-35 on cytokine production of peripheral and liver-resident CD8^+^ T cells from healthy individuals and non-viral hepatitis-related hepatocellular carcinoma. CD8^+^ T cells, which used in Figure 2, were stimulated with or without recombinant IL-35 and were cultured with PMA+ionomycin (peripheral) or AFP peptide (liver-resident) for 12 h. Cells were stained with anti-CD8 APC Cy7 and anti-IFN-γ APC for flow cytometry analysis. **(A)** Representative flow dots of IFN-γ secretion in CD8^+^ T cells with or without IL-35 stimulation were shown. **(B)** Percentage of IFN-γ-producing CD8^+^ T cells was compared prior to and post IL-35 stimulation. **(C)** IFN-γ expression in cultured supernatants was also compared prior to and post IL-35 stimulation. The columns indicated means, and the bars indicated standard deviation. Significances were determined using Student *t*-test or paired *t*-test.

CD8^+^ T cells, which were stimulated with or without recombinant IL-35, were cultured with PMA + ionomycin (peripheral) or AFP peptide (liver-resident) for 12 h. Representative flow dots of IFN-γ production in CD8^+^ T cells prior to and post IL-35 stimulation were showed in Figure 4A. IL-35 stimulation significantly reduced percentage of IFN-γ-producing CD8^+^ T cells in both peripheral and liver-resident cells (paired *t*-tests, all *P* < 0.05, Figure 4B). IFN-γ expression in cultured supernatants was also measured by ELISA. IL-35 stimulation significantly down-regulated IFN-γ expression (paired *t*-tests, *P* < 0.05, Figure 4C) expect in liver-resident CD8^+^ T cells from tumor tissues (paired *t*-tests, *P* = 0.117, Figure 4C).

### IL-35 Stimulation Suppressed Cytolytic and Non-cytolytic Activity of CD8^+^ T Cells From Non-viral Hepatitis-Related HCC

**Figure 5:**
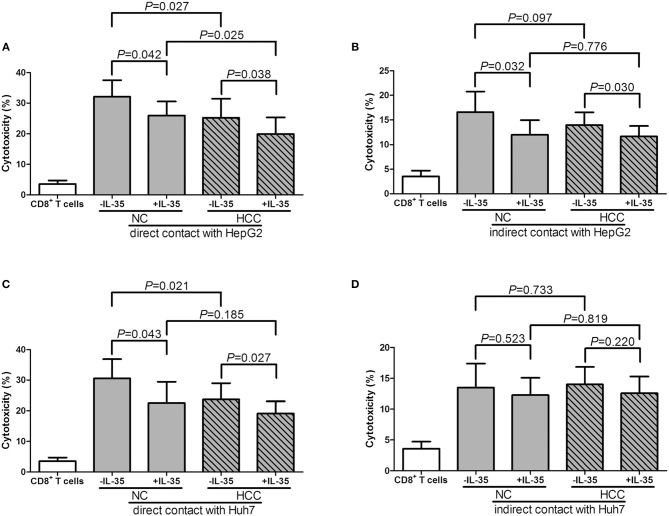
Cytotoxic effect of peripheral CD8^+^ T cells on HCC cell lines in direct and indirect contact co-culture systems. Peripheral CD8^+^ T cells were purified from HLA-A2 restricted healthy individuals (*n* = 7) and HCC patients (*n* = 12). 10^5^ of CD8^+^ T cells were stimulated with IL-35 for 6 h, and were co-cultured in direct or in indirect contact with 5 × 10^5^ of HepG2 cells or HLA-A2-expressing Huh7 cells in the presence of anti-CD3/CD28. Percentage of cell death was measured by LDH release. **(A)** CD8^+^ T cells were co-cultured in direct contact manner with HepG2 cells. **(B)** CD8^+^ T cells were co-cultured in indirect contact manner with HepG2 cells. **(C)** CD8^+^ T cells were co-cultured in direct contact manner with HLA-A2-expressing Huh7 cells. **(D)** CD8^+^ T cells were co-cultured in indirect contact manner with HLA-A2-expressing Huh7 cells. The columns indicated means, and the bars indicated standard deviation. Significances were determined using Student *t*-test or paired *t*-test.

10^5^ of purified peripheral CD8^+^ T cells from HLA-A2 restricted healthy individuals (*n* = 7) and HCC patients (*n* = 12) were stimulated with IL-35 for 6 h, and were co-cultured in direct contact or in indirect contact with 5 × 10^5^ of HepG2 cells or HLA-A2-expressing Huh7 cells in the presence of anti-CD3/CD28. The supernatants were harvested for further experiments 48 h post co-culture. In direct contact co-culture system, CD8^+^ T cells from healthy individuals revealed stronger cytolytic activity to target HCC cells than HCC patients, which presented as elevated percentage of cell death (Student *t*-tests, *P* = 0.027, Figure 5A; *P* = 0.043, Figure 5C). IL-35 stimulation significantly down-regulated percentage of cell death in both NC and HCC patients (paired *t*-tests, all *P* < 0.05, Figures 5A,C). In indirect contact co-culture system, IL-35 stimulation only reduced target HepG2 cell death in NC (paired *t*-test, *P* = 0.032, Figure 5B) and HCC patients (paired *t*-test, *P* = 0.030, Figure 5B), however, did not affect Huh7 cell death in either groups (paired *t*-tests, *P* = 0.523 and *P* = 0.220, respectively, Figure 5D). Cytokine productions were also investigated in the cultured supernatants. In direct contact co-culture system with HepG2 cells, IL-35 stimulation notably reduced the secretions of IFN-γ, IL-12p70, IL-6, IL-8, and TNF-α in CD8^+^ T cells purified from both NC and HCC (Table 4). However, only IFN-γ and IL-12p70 expression was down-regulated in response to IL-35 treatment in indirect contact co-culture system (Table 5). The cytokine production profile was similar in both direct and indirect contact system when peripheral CD8^+^ T cells were co-cultured with HLA-A2-expressing Huh7 cells (data not shown).

**Table 4:** Cytokine production by peripheral CD8^+^ T in direct co-culture with HepG2 cells (pg/mL).

	**NC (*****n*** **=** **7)**			**HCC (*****n*** **=** **12)**		
	**–IL-35**	**+IL-35**	***P*-value**	**–IL-35**	**+IL-35**	***P*-value**
IFN-γ	33.71 ± 8.48	21.49 ± 4.31	0.003	25.21 ± 8.03	17.78 ± 5.63	0.017
IL-1β	77.53 ± 18.37	69.85 ± 13.44	0.187	80.58 ± 11.74	76.24 ± 17.76	0.269
IL-10	N.D.	N.D.	–	N.D.	N.D.	–
IL-12p70	98.72 ± 28.61	32.89 ± 7.74	< 0.0001	84.24 ± 10.43	29.24 ± 9.79	< 0.0001
IL-6	109.4 ± 32.59	84.73 ± 23.11	0.0008	106.3 ± 20.47	77.10 ± 18.71	0.001
IL-8	123.1 ± 38.10	100.6 ± 28.71	0.006	117.8 ± 31.09	108.8 ± 17.29	0.047
TNF-α	2192 ± 563.3	1998 ± 348.3	0.035	1843 ± 667.0	1439 ± 385.9	0.037

*N.D., not detected. P-values were determined using paired t-tests*.

**Table 5:** Cytokine production by peripheral CD8^+^ T in indirect co-culture with HepG2 cells (pg/mL).

	**NC (*****n*** **=** **7)**			**HCC (*****n*** **=** **12)**		
	**–IL-35**	**+IL-35**	***P*-value**	**–IL-35**	**+IL-35**	***P*-value**
IFN-γ	27.20 ± 7.98	19.79 ± 6.18	0.0006	23.48 ± 2.94	17.18 ± 4.47	0.0071
IL-1β	56.19 ± 10.82	60.10 ± 8.87	0.727	57.99 ± 9.73	55.07 ± 11.23	0.591
IL-10	N.D.	N.D.	–	N.D.	N.D.	–
IL-12p70	84.19 ± 19.15	46.20 ± 11.78	< 0.0001	77.90 ± 16.74	55.62 ± 9.76	0.0001
IL-6	73.91 ± 20.99	71.39 ± 22.80	0.829	78.22 ± 17.98	70.89 ± 21.09	0.730
IL-8	90.41 ± 10.22	94.17 ± 16.49	0.241	89.72 ± 8.19	81.19 ± 17.65	0.221
TNF-α	983.1 ± 231.8	872.1 ± 102.4	0.0086	782.4 ± 109.3	712.4 ± 79.11	0.073

*N.D., not detected. P-values were determined using paired t-tests*.

**Figure 6:**
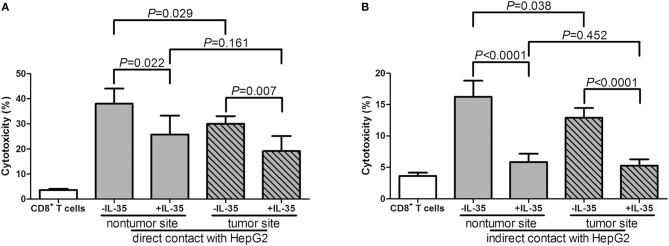
Cytotoxic effect of liver-resident CD8^+^ T cells on HCC cell lines in direct and indirect contact co-culture systems. Liver-resident CD8^+^ T cells were purified from tumor and non-tumor site of HLA-A2 restricted HCC patients (*n* = 5). 10^5^ of CD8^+^ T cells were stimulated with IL-35 for 6 h, and were co-cultured in direct or in indirect contact with 5 × 10^5^ of HepG2 cells in the presence of AFP peptide. Percentage of cell death was measured by LDH release. **(A)** CD8^+^ T cells were co-cultured in direct contact manner with HepG2 cells. **(B)** CD8^+^ T cells were co-cultured in indirect contact manner with HepG2 cells. The columns indicated means, and the bars indicated standard deviation. Significances were determined using Student *t*-test or paired *t*-test.

Furthermore, 10^5^ of liver-resident CD8^+^ T cells from tumor and non-tumor site of HLA-A2 restricted HCC patients (*n* = 5) were stimulated with IL-35 for 6 h, and were co-cultured in direct or indirect contact with 5 × 10^5^ of HepG2 cells in the presence of AFP peptide. The supernatants were harvested for further experiments 48 h post co-culture. CD8^+^ T cells from non-tumor site presented stronger cytotoxicity to target HepG2 cells than those from non-tumor site in both direct contact (Student *t*-test, *P* = 0.029, Figure 6A) and indirect contact co-culture system (Student *t*-test, *P* = 0.038, Figure 6B). IL-35 stimulation notably reduced the cytotoxicity of CD8^+^ T cells from both non-tumor and tumor site in both co-culture systems (paired *t*-tests, all *P* < 0.05, Figures 6A,B). Cytokine expression profile presented similar trends with those in peripheral CD8^+^ T and HepG2 cells co-culture systems. In direct contact co-culture system, IL-35 stimulation notably reduced the secretions of IFN-γ, IL-12p70, IL-6, IL-8, and TNF-α in CD8^+^ T cells purified from non-tumor and tumor site (Table 6). IFN-γ, IL-12p70, and TNF-α expression was down-regulated in response to IL-35 treatment in indirect contact co-culture system (Table 7).

**Table 6:** Cytokine production by liver-resident CD8^+^ T in direct co-culture with HepG2 cells (pg/mL).

	**Non-tumor site (*****n*** **=** **5)**			**Tumor site (*****n*** **=** **5)**		
	**–IL-35**	**+IL-35**	***P*-value**	**–IL-35**	**+IL-35**	***P*-value**
IFN-γ	47.84 ± 6.99	31.05 ± 8.17	< 0.0001	37.81 ± 10.26	26.63 ± 4.91	0.008
IL-1β	66.79 ± 15.27	63.83 ± 18.72	0.764	67.17 ± 13.61	66.93 ± 18.52	0.988
IL-10	N.D.	N.D.	–	N.D.	N.D.	–
IL-12p70	108.7 ± 27.35	75.93 ± 21.90	< 0.0001	93.40 ± 8.27	67.31 ± 14.27	< 0.0001
IL-6	98.16 ± 17.39	76.33 ± 19.76	0.007	89.31 ± 21.39	79.03 ± 9.90	0.021
IL-8	188.3 ± 54.41	141.3 ± 49.01	0.003	176.2 ± 55.31	109.7 ± 24.11	< 0.0001
TNF-α	4712 ± 996.1	3988 ± 780.0	0.001	3548 ± 648.2	2552 ± 700.4	< 0.0001

*N.D., not detected. P-values were determined using paired t-tests*.

**Table 7:** Cytokine production by liver-resident CD8^+^ T in indirect co-culture with HepG2 cells (pg/mL).

	**Non-tumor site (*****n*** **=** **5)**			**Tumor site (n=5)**		
	**–IL-35**	**+IL-35**	***P*-value**	**–IL-35**	**+IL-35**	***P*-value**
IFN-γ	42.85 ± 9.32	31.42 ± 7.14	< 0.0001	33.16 ± 13.70	23.74 ± 6.54	< 0.0001
IL-1β	56.59 ± 18.97	54.49 ± 17.02	0.683	59.56 ± 14.49	56.73 ± 16.23	0.764
IL-10	N.D.	N.D.	–	N.D.	N.D.	–
IL-12p70	98.13 ± 11.24	84.81 ± 25.14	0.041	85.45 ± 4.58	70.89 ± 29.41	0.037
IL-6	62.20 ± 23.74	69.88 ± 17.64	0.489	70.89 ± 11.85	66.72 ± 10.70	0.227
IL-8	85.07 ± 20.36	81.30 ± 17.61	0.371	73.67 ± 13.38	68.29 ± 13.10	0.181
TNF-α	3652 ± 391.2	2900 ± 135.2	0.0006	2812 ± 596.4	2584 ± 372.8	0.018

*N.D., not detected. P-values were determined using paired t-tests*.

## Discussion

In the present study, increased serum IL-35 was observed in patients with non-viral hepatitis-related HCC, especially in advanced stages, whereas effective anti-tumor therapy down-regulated IL-35 expression, indicating a close relationship between IL-35 and disease progression. Recombinant IL-35 stimulation inhibited cytolytic and non-cytolytic activity of peripheral and liver-resident CD8^+^ T cells purified from HCC patients, which presented as elevated cytotoxicity and proinflammatory cytokines production in both direct and indirect contact co-culture systems. This process was accompanied by reduction of perforin expression and interferon-γ production in CD8^+^ T cells. The current results indicated an important immunosuppressive property of IL-35 in non-viral hepatitis-related HCC.

IL-35 played a pivotal role in the pathogenesis of tumor, progression, and prognosis. Tumor derived IL-35 has been demonstrated to promote tumor progression and to be a prognostic indicator in non-small cell lung cancer (20), intrahepatic cholangiocarcinoma (21), colorectal cancer (22, 23), prostate cancer (24), and breast cancer (25). However, controversy remained as to the expression profile of liver-resident and peripheral IL-35 expression in HCC patients. Long et al. revealed a reduced expression of IL-35 in HCC tumor tissue, which correlated with worse histological grades, larger tumor size, identified microvascular invasion, and lymph node/distant metastasis (12). In contrast, immunohistochemistry study by Fu et al. indicated constitutively expressed IL-35 in HCC specimens was higher in advanced BCLC stage and larger tumor size, which was associated with HCC aggressiveness (11). A more recent study by Qiu et al. demonstrated that the increased circulating IL-35 expression was also an independent prognostic indicator in HCC patients (26). This was consistent with our current results, which was also showed an elevation of serum IL-35 concentration in non-viral hepatitis-related HCC, especially in BCLC stage D patients. More importantly, we also investigated that a decreased of serum IL-35 expression in HCC patients underwent operation (curative resection, liver transplantation, or TACE), although this was not aligned with the findings by Liao and colleagues, which indicated no statistical difference in IL-35 expression prior to or post-TACE in HCC patients (27). Thus, the role of increased serum IL-35 in non-viral hepatitis-related HCC needs to be further investigated.

Cancer evolution and progression always induced dysregulation or exhaustion of peripheral and tissue-resident CD8^+^ T cells, leading to the immunosuppressive tumor microenvironment and the dampening of anti-tumor immunity (28, 29). CD8^+^ T cells exhibited cytotoxic activity through two independent pathways, perforin/granzyme pathway and Fas/FasL interaction (30, 31). We found that perforin mRNA expression was down-regulated in peripheral CD8^+^ T cells from HCC patients, IL-35 stimulation reduced peripheral perforin mRNA in CD8^+^ T cells. Although there were no significant differences of perforin, granzyme, or FasL within liver-resident CD8^+^ T cells between non-tumor and tumor site, granzyme B mRNA within liver-resident CD8^+^ T cells from tumor site was reduced in response to IL-35 stimulation. The differential expression profile and responsiveness to IL-35 stimulation of perforin and granzyme in peripheral and liver-resident might indicate diverse mechanism of CD8^+^ T cells cytotoxicity to cancer cells. The exhausted markers, including PD-1 and CTLA-4, were increasingly expressed in CD8^+^ T cells in response to IL-35 stimulation, indicating another mechanistic insight for inhibitory molecules induction by IL-35. Furthermore, cytokine-induced cytotoxicity was another functioning cell death pathway, especially IFN-γ. Cultured CD8^+^ T cells exerted reduced IFN-γ production in HCC patients. These indicated an dysfunctional property of CD8^+^ T cells in non-viral hepatitis-related HCC. Recombinant IL-35 stimulation *in vitro* significantly reduced perforin mRNA expression in peripheral CD8^+^ T cells from both healthy individuals and HCC patients. IL-35 stimulation alone did not affect cytokines production by CD8^+^ T cells. This was partly due to the unexposure to antigens/peptides. More importantly, IL-35 robustly dampened IFN-γ secretion by AFP-specific liver-resident CD8^+^ T cells purified from both non-tumor and tumor site, further revealing the immunosuppressive characteristic of IL-35 to CD8^+^ T cells in non-viral hepatitis-related HCC.

The mechanism of antigen-specific CD8^+^ T cell-mediated cytotoxicity involved both cytolytic function of direct target cells killing and non-cytolytic activity of cytokine-mediated tumor rejection or viral clearance (18, 32). The *in vitro* direct and indirect co-culture model provided strong tools to investigate the cytolytic and non-cytolytic property of CD8^+^ T cells independently (18). By using this co-culture model, Yu et al. showed a weaker cytolytic activity of tumor-infiltrating CD8^+^ T cells to HT29 cell line in patients with colorectal carcinoma (33). Our previous study also revealed that HBV-specific CD8^+^ T cells not only destruct HBV-infected HepG2.2.15 cells, but also eliminated HBV replication in HepG2.2.15 cells, which induced by IFN-γ and TNF-α secretion without inducing cell injury (14). However, the previous study reported that CD8^+^ T cells from the blood of chronic hepatitis B patients were not able to exert indirect cytotoxic activity against HepG2.2.15 cells, which were the stable transfected HepG2 with HBV plasmid (14). Herein, the current results showed that CD8^+^ T cells from the bloods of normal controls or from the bloods or the liver of HCC patients were able to kill HepG2 cells in an indirect way, which seemed controversial from previous reports. In our opinion, this controversy might be due to the following reasons. Firstly, in the current study, CD8^+^ T cells were mainly stimulated with anti-CD3/CD28, which could induce total CD8^+^ T cell function. However, CD8^+^ T cells were stimulated with HBV core 18-27 peptide, which could only induce HBV core specific CD8^+^ T cell activity. The different functional CD8^+^ T cells numbers might be account for the difference in target cell death. Secondly, although CD8^+^ T cells isolated from different patients and controls were described to produce similar patterns of cytokines mediating cell death, TNF-α expression, which were secreted by anti-CD3/CD28 or AFP peptide-induced CD8^+^ T cells, was significantly higher in comparison with that induced by HBV peptide. The elevated TNF-α level might also contribute to the increased cell death in indirect co-culture system. Thirdly, it might be that different CD8^+^ T cell subsets are present in the different cultures. As described by Teng et al. (17), CD45RA naïve CD8^+^ T cells was reduced, while CD45RO memorial CD8^+^ T cells was elevated in HCC patients. We also found that 3–4% of CD8^+^CD25^+^CD127^dim/−^ regulatory cells could be detected in purified bulk CD8^+^ T cells (data not shown). IL-35 stimulation inhibited HBV-specific cytotoxic CD8^+^ T cells function and suppressed cytokine-mediated viral clearance in chronic HBV infection (14). However, the role of IL-35 to tumor antigen-specific CD8^+^ T cells was still not fully investigated, especially in patients with HCC. Thus, we set up the direct and indirect contact co-culture system in CD8^+^ T cells with target HCC cell lines in both AFP-specific and non-specific manners. Antigen non-specific peripheral CD8^+^ T cells presented limited cytotoxicity in HCC patients, which induced less target cells death and diminished proinflammatory cytokines production. AFP-specific liver-resident CD8^+^ T cells also showed weaker cytotoxicity in tumor site than those in non-tumor site. Recombinant IL-35 stimulation not only reduced target cells death, but also reduced proinflammatory cytokines secretion in direct and indirect contact co-culture system in both AFP-specific and non-specific manners. IL-35-mediated reduced target cell death in indirect contact co-culture system indicated the cytokine-induced tumor rejection, which was not comparable with non-cytolytic property of cytokine-mediated viral clearance without cell death. Further *in vivo* experiments are needed for the regulatory activity of IL-35 on antigen-specific CD8^+^ T cells in non-viral hepatitis-related HCC.

In summary, we found that elevated IL-35 expression in non-viral hepatitis-related HCC might reduce both cytolytic and non-cytolytic activity of antigen-specific and non-specific CD8^+^ T cells probably *via* repression of perforin expression. IL-35 might be considered to be one of the therapeutic targets for patients with HCC.

## Ethics Statement

This study was carried out in accordance with the recommendations of Ethics Committee of The Second Hospital of Jilin University with written informed consent from all subjects. All subjects gave written informed consent in accordance with the Declaration of Helsinki. The protocol was approved by the Ethics Committee of The Second Hospital of Jilin University.

## Author Contributions

LY, XS, and SJ performed the study. XS, SJ, QZ, and ZJ enrolled the patients. LY, XS, SJ, QZ, and ZJ analyzed the data, and prepared the manuscript. ZJ designed and supervised the study.

### Conflict of Interest Statement

The authors declare that the research was conducted in the absence of any commercial or financial relationships that could be construed as a potential conflict of interest.
